# Intracranial Manifestation of Melioidosis: A Case Report and Long-Term Follow-Up

**DOI:** 10.7759/cureus.12367

**Published:** 2020-12-29

**Authors:** Alba Corell, Aylin Yilmaz, Fawaz S Almotairi, Dan Farahmand

**Affiliations:** 1 Neurosurgery, Sahlgrenska University Hospital, Gothenburg, SWE; 2 Clinical Neuroscience, Sahlgrenska Academy, University of Gothenburg, Gothenburg, SWE; 3 Infectious Diseases, Sahlgrenska University Hospital, Gothenburg, SWE; 4 Infectious Diseases, Sahlgrenska Academy, University of Gothenburg, Gothenburg, SWE; 5 Neurosurgery, King Saud University, Riyadh, SAU

**Keywords:** burkholderia pseudomallei, epidural abscess, neurosurgery, treatment, melioidosis

## Abstract

Primary neurological melioidosis is rare with fewer than 50 cases reported world-wide. We report the first documented case of primary neurological melioidosis in Sweden, a 32-year old male who previously lived in Thailand for six years and recently moved to Sweden. He presented with headache, irritability and lack of concentration. Investigation with computerized tomography (CT) and subsequent magnetic resonance imaging (MRI) showed epidural fluid that was interpreted as a chronic epidural hematoma. He underwent surgical evacuation of the epidural collection that was found to be a white collection mixed with pus and bacterial culture results were positive for *Burkholderia pseudomallei*.

## Introduction

Melioidosis is a common tropical infection in Southeast Asia, northern Australia, South Asia, and China caused by the bacterium *Burkholderia pseudomallei*, a Gram-negative and highly pathogenic bacillus in soil and fresh surface water [1]. Transmission can occur through inhalation, aspiration, percutaneous inoculation, and sometimes through ingestion. Neurological manifestations in association with melioidosis are uncommon and have been reported to be approximately 3-7% in previous case reports and series [2-5]. Furthermore, primary central nervous system (CNS) involvement is rare and to the best of our knowledge has been scarcely reported in the literature [6]. The mortality rate of neurological melioidosis is high and has been reported to be between 9 and 30% [3, 7]. Early diagnosis of neurological melioidosis decreases associated mortality. We report the first case of neurological melioidosis diagnosed in Sweden with intracranial involvement, review the relevant literature and highlight the importance of early diagnosis and management of this rare infection. 

## Case presentation

History

The patient is a 32-year old Caucasian male with diabetes mellitus type 1, intermittent alcohol consumption, and drug abuse. He had lived in Thailand for six years and returned to Sweden two years ago. He had been involved in three mild motorcycle accidents in Thailand but no neuroradiological investigations had been done. Furthermore, he had previously tested negative for both HIV and hepatitis.

Presenting symptoms

**Figure 1:**
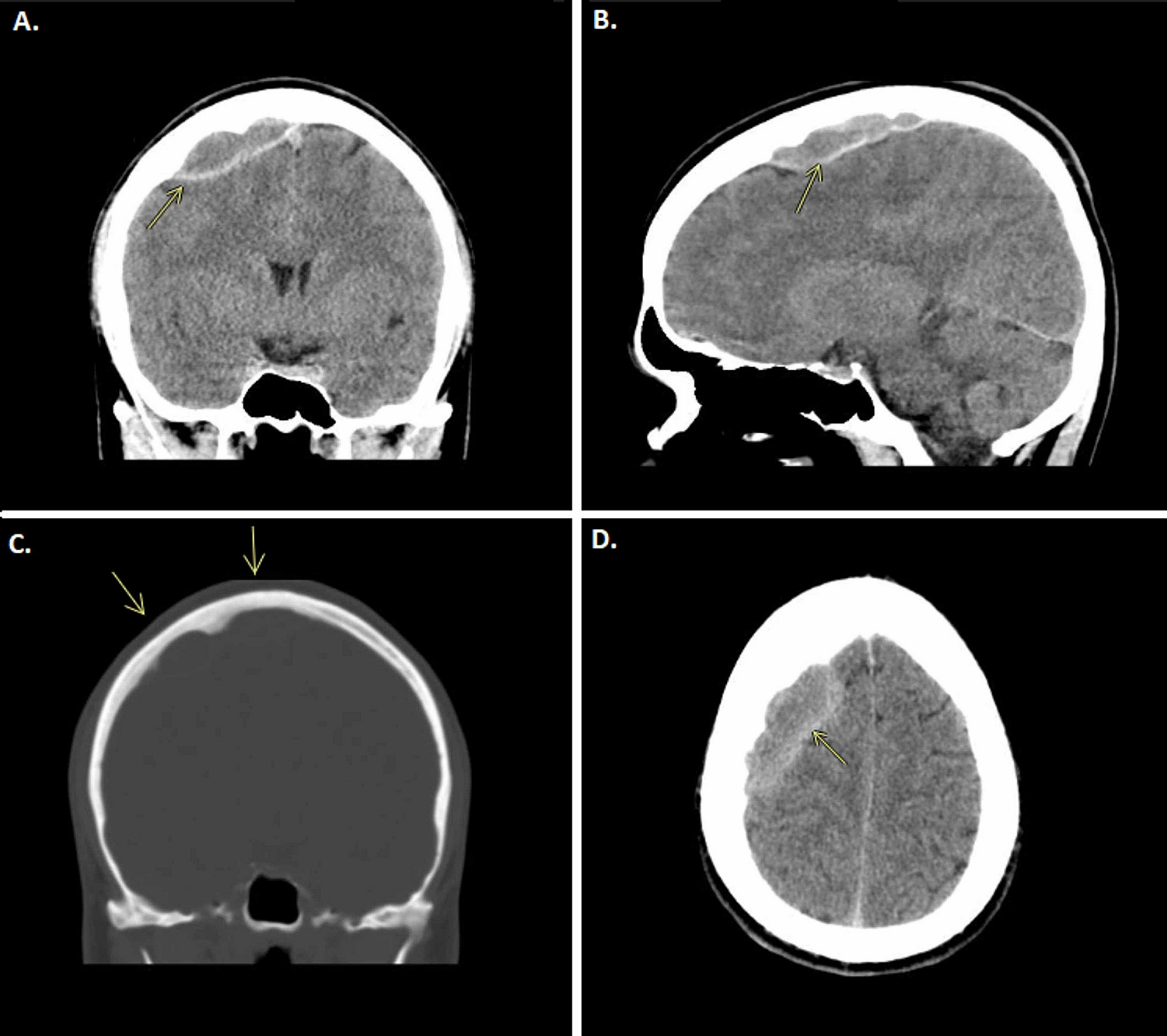
CT scan without contrast of the brain in coronal, sagittal and axial plane. AB, D; CT without contrast, showing the epidural collection of the right frontoparietal area, see arrows. C; CT with bone window, arrows pointing at scalloping in the adjacent neurocranium and suspected osteomyelitis.

He complained of recurrent episodes of frontoparietal headache for the last year that was relieved by over-the-counter analgesics. His symptoms worsened during the last month before first admission with increased sensitivity to light, decreased ability to concentrate, and irritability. He sought medical advice at the primary health care centre and was referred to get a computerized tomography (CT) of the brain. This showed a large epidural mass at the right frontoparietal area that crossed the midline and compressed the sagittal sinus (Figure 1A, B and D). The adjacent neurocranium showed scalloping of the inner table (Figure 1C). Initial radiological diagnosis was an organised chronic epidural hematoma and accordingly, he was referred to the emergency department at Sahlgrenska University Hospital.

**Figure 2:**
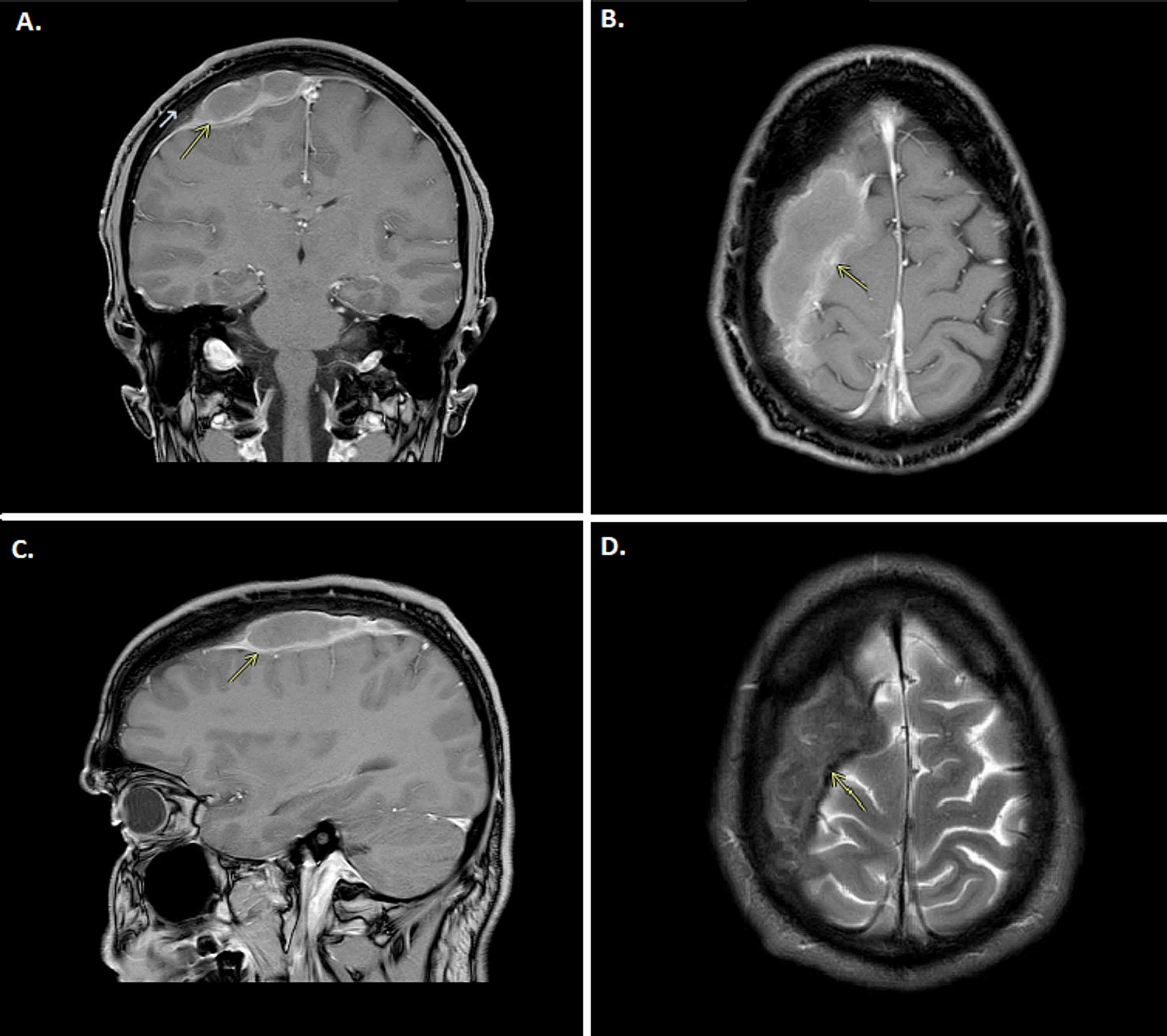
MRI scan of the brain. ABC; T1-weighted image with contrast, coronal, axial and sagittal view showing a mass with high signal intensity and peripheral contrast enhancement. D; T2-weighted image, axial plane that shows a low signal intensity of the collection.

At initial examination, he was fully oriented and had intact higher cognitive functions. The detailed neuro-ophthalmological and neurological examination were unremarkable. Laboratory analysis showed C-reactive protein (CRP) 9 mg/L, leucocyte count 5.7 x 10^9^, and normal haemoglobin and creatinine levels. Magnetic resonance imaging (MRI) with and without contrast was performed, which showed high signal intensity on the T1-sequence (Figure 2A, B and C) and low signal intensity on the T2 sequence (Figure 2D), and signs of heterogeneous diffusion restriction. Initial MRI report was also interpreted as an old, organized epidural hematoma. There was a peripheral contrast enhancement in the mass. At initial examination, he was fully oriented and had intact higher cognitive functions. The detailed neuro-ophthalmological and neurological examination were unremarkable. Laboratory analysis showed a CRP of 9 mg/L, leucocyte count 5.7 x 10^9^, and normal haemoglobin and creatinine levels. Magnetic resonance imaging (MRI) with and without contrast was performed, which showed high signal intensity on the T1-sequence (Figure 2A-C) and low signal intensity on the T2 sequence (Figure 2D), and signs of heterogeneous diffusion restriction. Initial MRI report was also interpreted as an old, organized epidural hematoma. There was a peripheral contrast enhancement in the mass.

Course of care

The patient was transferred to the neurosurgical department and underwent evacuation of the epidural collection. A craniotomy centralized over the mass was performed with a size of 57 x 70 mm. This turned out to be a white foul-smelling tissue of medium thickness mixed with pus. Furthermore, a well-organized white solid epidural mass was found. It was excised and sent for histopathology and bacterial culture. The dura was left intact and the bone flap was replaced using titanium plates. No perioperative complications were reported and postoperatively he was started on prophylactic cefuroxime. At the second postoperative day, he developed fever at 38.6^0^C, general malaise, and tachycardia. Laboratory analysis showed CRP 74 mg/L, leucocyte count 7.0 x 10^9^, and negative bacterial cultures in both blood and urine. Thereafter, his antibiotic regimen was changed to the carbapenem meropenem in meningitis dosage (2 g every eight hours). He started to improve clinically at day 4-5 after surgery and CRP gradually decreased. Bacterial culture from the epidural collection showed growth of *Burkholderia pseudomallei*, MIC values (mg/L) was 1 for meropenem, 2 for ceftazidime, 1.5 for amoxicillin/clavulanic acid, 6 for trimethoprim-sulfamethoxazole (TMP-SMX) and 1 for doxycycline. He was admitted to the Department of Infectious Diseases, Sahlgrenska University Hospital, for further treatment. Seven days postoperatively, he developed signs of wound infection and CT of the brain showed a highly suspected intracranial abscess in the previously operated area. He underwent surgical evacuation of the abscess and removal of the bone flap and continued with meropenem with the addition of TMP-SMX for eight weeks. After this period of time, the previously healed wound spontaneously opened and pus emptied out. The patient was clinically stable with no other symptoms or signs. CT scan showed an epidural fluid collection. New samples for bacteriology was taken and no surgical intervention was performed. The samples showed no growth of *Burkholderia pseudomallei*, but confirmed presence of DNA by PCR. The patient continued the antibiotic regimen stated previously for six months, with some interruptions of TMP-SMX because of adverse reactions such as fever and nausea.

One month after cessation of antibiotics he underwent a new CT scan with and without contrast with no apparent signs of infection, and he was scheduled for cranioplasty with acrylic plate after three months. However, the immediate preoperative CT scan with contrast showed a contrast-enhancing fluid collection epidurally. Surgical evacuation was performed without any cranioplasty, and the samples for bacteriology again showed growth of bacteria *Burkholderia pseudomallei*, MIC values (mg/L) was 0.5 for meropenem, 1 for ceftazidime, 2/2 for amoxicillin/clavulanic acid, 1/19 for TMP-SMX. He was treated using intravenous meropenem for two months with concomitant and adjuvant oral treatment with TMP-SMX for a total of 8 months. This time total length of treatment was eight months and the patient tolerated TMP-SMX much better. Three weeks after the antibiotic regimen was finished a new CT scan was performed, which showed total regression of the abscess and no contract-enhancement. This was followed three months later by a cranioplasty with acrylic plate. The preoperative CT scan with contrast did not show any signs of infection, nor was there any suspicion regarding infection during the surgery.

## Discussion

Intracranial melioidosis is a very rare finding in Scandinavia and the western hemisphere with only a few cases previously diagnosed in Europe [6,8,9]. The initial investigations with CT and MRI scans in the present case pointed towards chronic epidural hematoma, itself an atypical neurosurgical condition [10].

The most common risk factors for developing melioidosis are diabetes, alcohol use, drug abuse, chronic lung disease, and chronic renal failure [2,3]. Clinical manifestations of melioidosis are non-specific and diverse. However, primary cutaneous melioidosis in children and pneumonia in adults are the most common reported clinical presentations [4]. Previous reports of neurological manifestations of melioidosis included limb weakness, cerebellar signs, and cranial nerve dysfunction as well as meningitis-like symptoms such as fever and neck stiffness [5,6,8]. Our patient had several predisposing factors for melioidosis: he lived in an endemic area in Thailand and had some of the disease risk factors (diabetes, drug abuse, and increased alcohol consumption). Furthermore, he had milder symptoms as compared to previous reports of neurological melioidosis and initially no signs of infection.

Initial investigations in patients who were found later on to have neurological melioidosis included CT and MRI that pointed in most of the cases toward nonspecific brain abscess [6]. Moreover, similar to our case, epidural location of melioidosis with bony changes consistent with osteomyelitis were found in a 45-year old female rice farmer who presented with a fluctuant skull mass [11]. The typical MRI features of empyema are usually hyperintensity on T1-sequence with strong peripheral contrast enhancement and iso- or hyperintensity on T2-sequence [12]. On the other hand, MRI features of chronic hematoma shows high signal intensity on T1-sequence and low signal intensity on T2-sequence that is similar to the present case [13], making correct neuroradiological diagnosis challenging. It has been found that growth of *Burkholderia pseudomallei* in blood and urine cultures is usually associated with poor prognosis, making this an important part of initial investigations [14].

How *Burkholderia pseudomallei *spreads to the CNS is still debated. It has been suggested that it enters the CNS by colonizing the respiratory epithelium in the nasal cavity and that it migrates along the olfactory and trigeminal nerves, thereby by-passing the blood-brain barrier into the cranial cavity [15]. It has been also speculated that bacteria enter the CNS through secondary dissemination from the lungs [8]. Moreover, the present case had previously mild head injuries and a direct spread to the intracranial space through a fracture in the skull adjacent to an open wound could explain the dissemination into CNS.

*Burkholderia pseudomallei* are intrinsically resistant to several antibiotics (such as penicillin, first- and second generation cephalosporins and aminoglycosides). Main therapeutic alternatives include meropenem, ceftazidime and TMP-SMX depending on the phase of treatment [7,16,17]. In the initial intensive phase in patients with CNS infection, high-dose meropenem (2g IV every eight hours) is recommended. The intensive phase with intravenous antibiotics is given for at least four to eight weeks in patients with neurologic melioidosis and in some other cases (for example prolonged critical illness, osteomyelitis, and septic arthritis), after the intensive phase, there is a longer period with eradication therapy to prevent relapse of melioidosis. The first choice is oral TMP-SMX alone. Doxycycline is an alternative when TMP-SMX cannot be used. The dosage of TMP-SMX for our patient (74 kg) was 320 mg of the trimethoprim component (two double strength tablets) orally twice daily. The duration of the eradication phase for neurologic melioidosis is six months.

Neurosurgical intervention in neurological melioidosis in previously reported cases included biopsy and evacuation of abscess and only one patient underwent evacuation of epidural collections and craniectomy [6,8,18]. We managed our patient in line with previous recommendations using meropenem after surgical evacuation and initial replacement of bone flap that later was removed due to new abscess formation. He was treated with intravenous meropenem and peroral TMP-SMX, the latter during six months with some interruptions due to adverse events. Control with CT scan with and without contrast two months postoperative showed abscess formation under the skin flap which was treated with continued antibiotics. Follow up CT scan with and without contrast after the discontinuation of the antibiotics showed regress of the abscess. However, when he presented to the neurosurgical department for cranioplasty, the preoperative CT scan with contrast showed relapse of the infection which was treated surgically. After a prolonged antibiotic regimen of up to 8 months, he was treated with cranioplasty and there were no signs of a new infection radiologically or perioperative.

*Burkholderia pseudomallei* is a Gram-negative, facultatively anaerobic and motile bacterium [19]. Treatment strategies includes dual antibiotic therapy, initially intravenous meropenem or ceftazidime, followed by eradication phase with TMP-SMX [20]. Prolonged treatment is usually recommended in patients with neuro-melioidosis. Our patient had difficulties following through the oral treatment with TMP-SMX due to adverse events he skipped doses, although the exact amount was unknown. The second time, he tolerated the antibiotic without interruption of therapy.

## Conclusions

Melioidosis was found intracranially in the epidural space in a 32-year old patient with diabetes type I who lived in Thailand for six years. This is one of few case reports of intracranial melioidosis in Europe. The patient in the present report did not suffer severe neurological manifestations and had no signs of infections upon presentation and had excellent outcome after surgical evacuation and treatment with meropenem and trimethoprim-sulfamethoxazole. We believe that the reported management of this case could give directions to early suspect intracranial melioidosis especially in travelers from endemic areas to western countries and Scandinavia.
